# Eco-Friendly, High-Ductility Slag/Fly-Ash-Based Engineered Cementitious Composite (ECC) Reinforced with PE Fibers

**DOI:** 10.3390/polym14091760

**Published:** 2022-04-26

**Authors:** Eskinder Desta Shumuye, Jie Liu, Weiwen Li, Zike Wang

**Affiliations:** 1 Guangdong Provincial Key Laboratory of Durability for Marine Civil Engineering, Shenzhen Durability Centre for Civil Engineering, College of Civil and Transportation Engineering, Shenzhen University, Shenzhen 518054, China; eskdes@szu.edu.cn (E.D.S.); 2060471051@email.szu.edu.cn (J.L.); 2 Department of Construction Technology and Management, Hawassa University Institute of Technology, Hawassa 05, Ethiopia; 3 School of Mechanics and Safety Engineering, Zhengzhou University, Zhengzhou 450001, China; zkwang@zzu.edu.com

**Keywords:** ECC, slag, tensile performance, crack characteristics, microstructure, PE

## Abstract

Engineered cementitious composites (ECCs) are a special class of ultra-ductile fiber-reinforced cementitious composites containing a significant amount of short discontinuous fibers. The distinctive tensile strain-hardening behavior of ECCs is the result of a systematic design based on the micromechanics of the fiber, matrix, and fiber–matrix interface. However, ECCs require extensive cement content, which is inconsistent with the goal of sustainable and green building materials. Consequently, the objective of this study is to investigate the mechanical performance of slag/fly-ash-based engineered cementitious composites (ECCs) reinforced with polyethylene (PE) fiber under axial compressive loading, as well as direct tensile and flexural strength tests. The composites’ microstructure and mineralogical composition were analyzed using images obtained from scanning electron microscopy (SEM), X-ray energy diffraction spectroscopy (EDS), X-ray powder diffraction (XRD), and X-ray fluorescence (XRF). The experimental results reveal that a slag-containing composite mixture shows strain-hardening behavior and comparable ductility properties to those of fly-ash-based composite mixtures. A ternary system of binder materials with 5% and 15% slag can increase the compressive strength of ECC by 3.5% and 34.9%, respectively, compared to slag-free ECC composite. Moreover, the microstructural results show that the slag-based cementitious matrix has a more closely cross-linked and dense microstructure at the matrix–aggregate interface. In addition, the concentration of particles on the surface of the fibers was higher in the slag-based cementitious composites than in the fly ash-based composite. This supports the concept that there is a stronger bonding between the fibers and matrix in the slag-based cementitious matrix than in fly-ash-based matrix.

## 1. Introduction

Engineered/strain-hardening cementitious composites (ECCs/SHCCs) are a type of high-performance fiber-reinforced cementitious composite with good multi-cracking behavior and tensile ductility [1,2]. ECCs have a substantially higher tensile strain capacity (usually 2–5%) than regular concrete or conventional fiber-reinforced concrete [3,4]. Even after being subjected to a substantial load, the crack width of ECCs remains relatively small, usually less than 60 mm [5]. This property of ECCs is a result of the controlled matrix fracture integration and consistent fiber bridging provided by the microfibers in the composite structure [6,7]. In the mix design of ECCs, coarse aggregates are excluded, resulting in a higher cement concentration and fiber content than those of conventional concrete [8], which incurs a higher cost and more shrinkage [9,10]. In addition, a high cement content results an increase in greenhouse gas emissions [11].

Researchers around the world have conducted various studies to develop composite materials with better performance, high strength, and longer lifespan. Currently, researchers are working to improve not only the strength but also to enhance the durability of composite materials by introducing supplementary cementitious materials and fibers into cementitious composite matrix. As a result of applied stress, cracks start to appear on the composite structural component, and the fibers create a bridging effect along the damage surface, hindering further crack propagation [12]. Additionally, the incorporation of natural fiber into the cementitious composite is also advantageous due to the availability of fibers in a variety of forms with a wide range of diameters, shapes, textures, and lengths [13,14]. However, compared to synthetic fiber-reinforced composites, such as polypropylene (PP), polyvinyl alcohol (PVA), and polyethylene (PE), the low strength of ECCs limits their application in products such as residential interior design materials, automobile and appliance parts, furniture, electrical equipment, conduits, etc. [15]. According to the literature, the most widely used supplementary cementitious materials (SCMs) during the production of ECCs are fly ash [16], slag [16,17], and rice husk [18,19]. Recently, limestone and calcined clay have also been proposed as new economical and sustainable cementitious materials [20,21], which, when used together with Portland cement, contribute to the properties of both fresh and hardened concrete through the process of pozzolanic or/and hydraulic reaction [22]. Pozzolanic materials are generally composed of the same oxides as clinker but in distinct proportions [23]. Considering the common notation for the cement hydration process, the main oxides are C (CaO), S (SiO_2_), A (Al_2_O_3_), F (Fe_2_O_3_), $ or S(SO_3_), and H (H_2_O). The final products of pozzolan–lime reactions are similar to those of Portland cement hydration, CSH, C_4_AH_13_, C_8_AFH_26_, and C_4_A$H_12_ [23,24,25]. The pozzolanic activity can be described in simplified terms by the following reactions [26,27].

(1) C+H →CH

(2) $$2S+3CH\;\to \;C_{3}S_{2}H_{3}$$

(3) $$A+F+8CH+18H\;\to \;C_{8}AFH_{26}$$

The calcium hydroxide required for the reactions in Equation (2) through Equation (3) comes from the pozzolanic material itself, from the hydration product of Portland cement (pozzolanic activity), or from self-pozzolanic activity, as shown in Equation (1). The hydration of various phases in OPC is given in [25]:

(4) $$C_{2}S+H\to CSH+2CH$$

(5) $$C_{3}S+H\to CSH$$

The formation of C–H and C–S–H is the result of the hydration of C_3_S and C_2_S. C–S–H is the main product of cementitious materials that imparts strength to cement-based materials [28,29]. The hydration products of C_3_A and C_4_AF do not contribute significantly to the strength development of hardened cement. The C–H product, which comes from the cement hydration, provides the required alkalinity to the hardened cement in the early stages of concrete curing. 

C–H formed during OPC hydration reacts with active silica and alumina in the presence of water to produce additional CSH and calcium aluminate hydrate (C–A–H), as shown below [25].

(6) CH+S+H→CSH

(7) CH+A+H→CAH

The C–H produced during the hydration of Portland cement reacts with slag. In blended cements, the consumption of C–H during the pozzolanic reaction results in a lower amount of C–H and an increase in C–S–H [30,31]. By weakening the Si–O, Al–O, and Al–O–Si covalent bonds, C–H serves as an activator for the solvation of ions containing Si and Al. The addition of slag cement induces the production of very fine hydration products, which refine the pores.

Studies from several concrete associations and producers around the world, particularly in Europe and North America, have indicated that high-quality Class F and Class C fly ash appears to be in limited supply [32]. This is because many countries throughout the world are rapidly shifting toward green and sustainable energy production, with coal facilities likely to be phased out entirely soon [33]. On the contrary, the supply of slag is expected remain sustainable. The use of slag in the production of ECCs also reduces waste and enhances the durability and mechanical properties of the composites. In comparison to fly ash, slag has more consistent chemical and physical properties. Partially or completely substituting slag in conventional cement-based products improves flexural strength and tensile strain capacity, as well as offering better resistance to sulfate attack and chloride penetration, as shown in previous studies [34,35,36,37]. Other studies showed that slag-blended ECCs develop a strong matrix, especially at early stages of aging [11]. Wu et al. [38] reported that a higher proportion of MgO in ECC materials resulted in a higher tensile strength, moderate crack distribution, and better water impermeability. However, there is a slight reduction in compressive strength and elastic modulus in the composite matrix. Similar studies by Zhu et al. [11] found that incorporation of mineral admixtures (FA and SL) can also result in strain-hardening behavior, as well as increased compressive strength and tensile capacity, especially during the early age of hydration. In the same study, the authors focused on the combined effect of fly ash and slag on the mechanical properties of ECC but did not include the mineralogical and microstructural influences of slag replacement on the engineered cementitious composite matrix. Choi et al. [39] also mentioned that the use of fly ash could improve the tensile strength of composites and the ductility of ECCs. The research results indicate that ECCs with fly ash tend to have reduced compressive strength in the early stage; however, flexural test results show better ductility. Additionally, Zhu et al. [40] reported that the compressive strength of ECCs with fly ash and slag can be reduced by up to 40% and 14%, respectively. For the ternary system of binder materials with replacement of 70% of cement, the combination of fly ash and slag can maintain excellent ductility of ECCs, as well as improved matrix strength. 

Because most studies on ECCs have focused on physical and mechanical properties, only limited researched has been conducted on the microstructural alteration and mineralogical composition of ECC mixtures with various slag replacements. The purpose of the present study is to investigate the microstructural alteration and mineralogical changes in addition to studying the ductility and compressive strength of four different ECC mixtures in a ternary material binder system with different supplementary cementitious materials (FA, Slag, and OPC). The water-to-cementitious materials ratio (w/c_m_) is kept 0.3 for all ECC mixtures. Consequently, in this study, we investigated the effect of various slag/fly ash replacements on the compressive strength, direct tensile strength, three-point bending strength, microstructural alterations, and changes in mineralogical composition of the ECC mixtures. 

## 2. Materials and Methods

### 2.1. Raw Materials and Mix Proportion

Class F fly ash (FA), Type I Portland cement (OPC), slag (S), water (W), high-range water reducer (HRWR), and polyethylene (PE) fibers were used to prepare slag/fly-ash-based ECC specimens. Table 1 presents the ECC mixture compositions. The mixing process used in this study is based on standard ECC mixing procedures reported in the literature [41]. The chemical compositions of fly ash and slag and the physical/mechanical properties of PE fibers are summarized in Table 2 and Table 3, respectively. 

For simplicity, specimens were labeled based on slag (S) and fly ash (F) replacement percentage. For example, for the 0% slag and 60% fly ash replacement, SF-0-60; for 5% slag and 55% fly ash, SF-5-55; for 10% slag and 50% fly ash, SF-10-50; and for 15% slag and 45% fly ash, SF-15-45. 

### 2.2. Specimen Preparation

Specimen preparation followed the recommendations of Huang et al. (2013) [3] for ECC mixing procedures. All solid ingredients (Portland cement, slag, and fly ash) were mixed in a 40 L Pan mixer for 3–4 min without water. The solid ingredients were then mixed with tap water and HRWRA for 5 min to achieve a flowable and homogeneous slurry based on the mix design. Subsequently, the slurry was cast into cubical, prism, and dog-bone-shaped molds and vibrated on an electromagnetic vibration table for 5 min. Before demolding, all specimens were covered with a plastic sheet for 24 h.

### 2.3. Compressive Strength

Mechanical property tests of these ECC specimens with various percentages of slag and fly ash were carried out according to modified GB 50081-2003 [42]. The compressive strength of concrete (100 mm cube) was tested at a loading rate of 0.5 MPa/s using an MTS YAW6206 electrohydraulic servo pressure-testing machine with a capacity of 2000 kN, manufactured by MTS systems (YAW4206, Shenzhen, China) CO. LTD. For each set of slag/fly-ash-based ECC mixes, a set of three specimens was tested 7, 14, and 28 days after casting.

### 2.4. Tensile Strength

**Figure 1:**
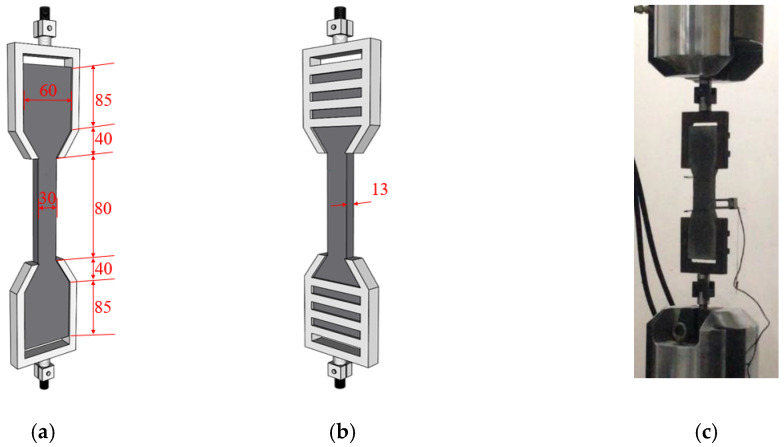
(**a**,**b**) Geometry and (**c**) test setup of uniaxial tensile test on dog-bone specimens (unit: mm).

The tensile stress and strain properties of the slag/fly-ash-based ECC specimens were determined using uniaxial tensile tests on the dog-bone shaped specimens. The use of dog-bone specimens enables cracks to be formed within the gauge length as a result of their smaller cross-sectional area than that of the end regions. ECC specimens with dimensions of 80 mm (length) × 30 mm (width) × 13 mm (depth) were used for these tests. The tests were performed using an ETM 305D electrohydraulic servo pressure-testing machine with a capacity of 300 kN, manufactured by MTS systems (E45.605 MTS, Shanghai, China) CO. LTD. A loading rate of 0.4 mm/min was used for all experiments. The detailed test setup for measurement of tensile strain by linear variable differential transformers (LVDTs) is shown in Figure 1.

Three specimens of each ECC mix were tested until final failure to obtain the tensile properties after 7 and 28 days of aging. The reference results (0% slag) were used as the control value to evaluate the effect of slag. The test setup is displayed in Figure 1. The tensile stress–strain curves were recorded, and the ultimate tensile strength, stiffness, and tensile strain capacity were determined [19]. 

### 2.5. Flexural Bending Test

**Figure 2:**
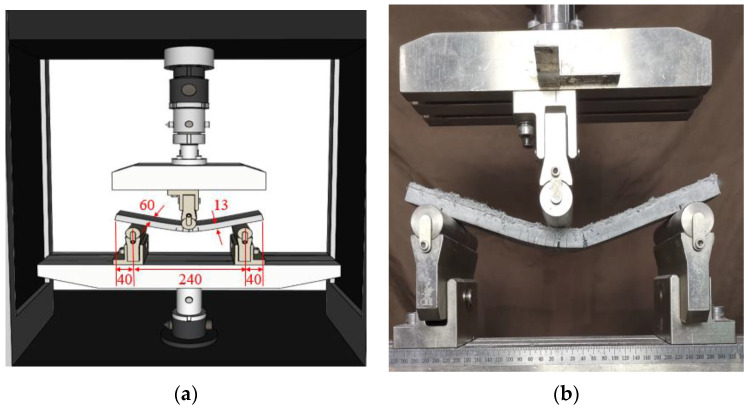
(**a**) Geometry and (**b**) test setup of three-point bending test specimens (unit: mm).

The flexural properties of slag/fly-ash-based ECC specimens were measured by three-point bending tests performed on a prism specimen using an MTS C64.305 hydraulic servo universal tension and compression testing machine manufactured by MTS systems (China) CO. LTD. The test was performed on an ECC specimens with a gauge dimensions of 320 mm (length) × 60 mm (width) × 13 mm (depth) and a loading rate of 0.4 mm/min. The experimental test setup for measurement of the flexural strength is shown in Figure 2. This test was performed on specimens of each mix after 7 and 28 days of curing time. For each set of slag/fly-ash-based ECC mixes, a set of three specimens was tested, and an average value was determined.

### 2.6. Microstructure and Mineralogical Characterization

**Figure 3:**
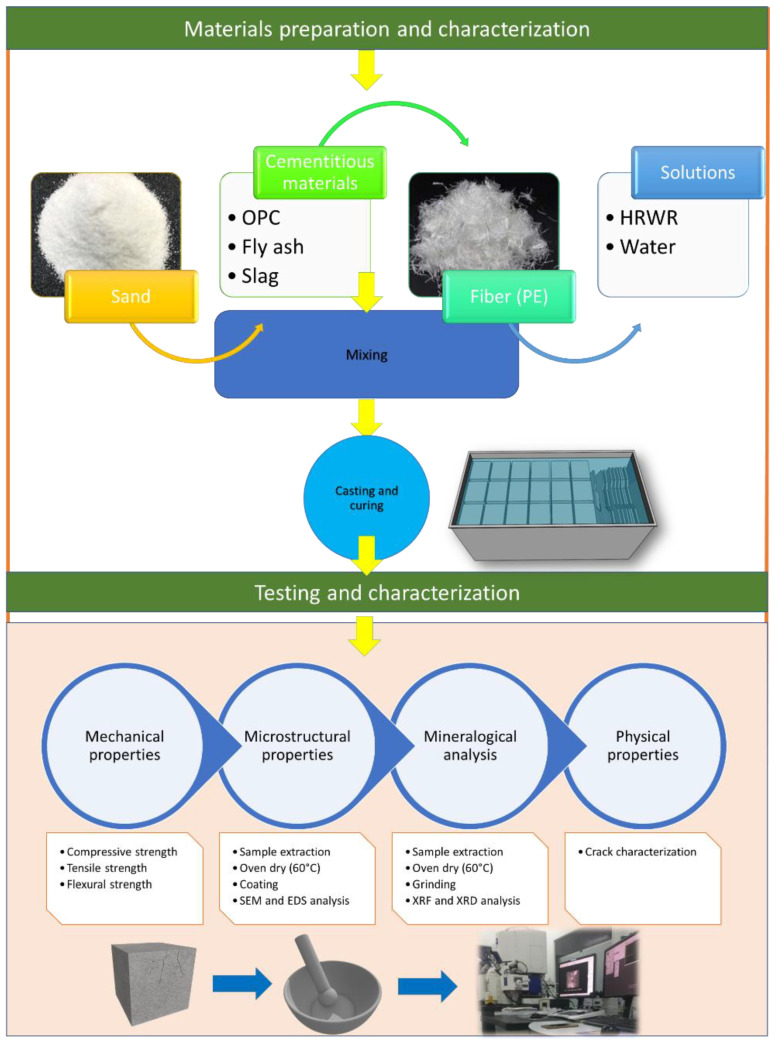
Overall flowchart of the methodology.

To study the microstructural qualities of ECCs, small fragments were carefully extracted from the selected dog-bone specimens using a rubber hammer. The microstructure and mineralogical characteristics were analyzed using a Hitachi SU8020 scanning electronic microscope (SEM) with energy-dispersive spectroscopy (EDS) and X-ray fluorescence (XRF). After drying, each sample was coated with a platinum layer to prevent charge collection on the surface [43,44]. The figure given in Figure 3 represents an overall experimental set up.

## 3. Results and Discussion

### 3.1. Compressive Strength

**Figure 4:**
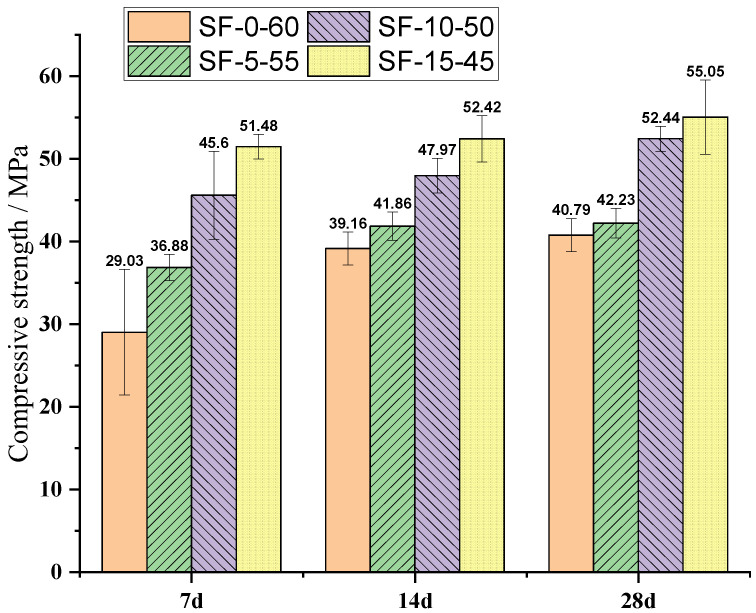
Compressive strength of slag/fly-ash-based ECCs at different curing stages.

The compressive strength test results of slag/fly-ash-based ECC mixtures with different slag contents at curing times of 7, 14, and 28 days are given in Figure 4. For each ECC mixture, the average of three specimens is presented. As shown in Figure 4, the replacement of fly ash by slag generally resulted the compressive strength of the slag/fly-ash-based mixtures being higher than those of the fly-ash-based mixtures under uniaxial compressive loading. Similar findings were reported in [17,45]. After 7 days of curing time, the compressive strengths of SF-15-45, SF-10-50, and SF-5-55 were 77.3%, 57.0%, and 27.0%, respectively—higher than that of SF-0-60 specimens containing only fly ash, highlighting the more gradual pozzolanic reaction of the fly ash. The addition of varying amounts of slag (Table 1) also significantly increased the compressive strength after 28 days of curing (40.79 to 55.05 MPa). After 28 days of curing time, the compressive strength of the slag/fly-ash-based mixture was 3.5%, 28.5%, and 34.9% for SF-5-55, SF-10-50, and SF-15-45 ECC mixtures, respectively, when compared to that of the purely fly-ash-based ECC mixes (SF-0-60) [46]. 

In addition, Razavi et al. (2021) [44] found that the compressive strength increased considerably from 35 Mpa on day 28 for a zeolites-to-cement replacement ratio of 1.2, 1.6, and 2. These results suggest that the addition of pozzolanic materials may have delayed the formation of portlandite and calcite in the matrix at early stages, improving bond quality [46]. Similarly, at a later stage, it might have a certain effect on the reduction in portlandite amount, which results in the same effect. This is further considered in the section discussing microstructural analysis.

### 3.2. Tensile Strength

**Figure 5:**
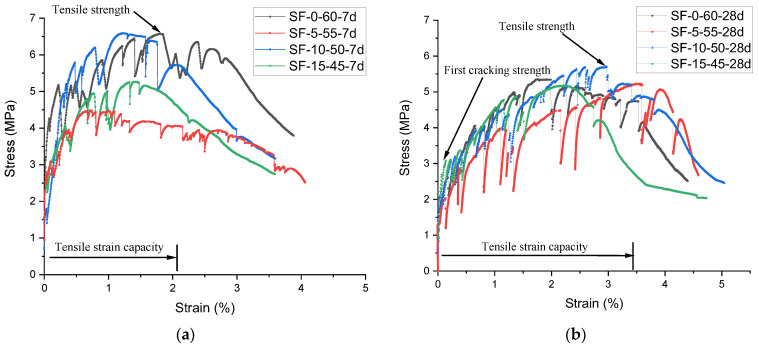
Stress–strain curves of slag/fly-ash-based ECC mixtures: (**a**) 7 days (**b**) 28 days. Curves represent the average of measurements of three separate specimens.

Table 4 summarizes the mechanical properties of SF-0-60, SF-5-55, SF-10-50, and SF-15-45, and their tensile stress–strain curves are shown in Figure 5. Each curve is the average of measurements of three specimens of the same composition. The average ultimate strain was used as the final strain value, so the three samples of each composite mix to had the same strain ranges. The average stress–strain curve of slag-free composite (SF-0-60) was used as a point of reference for comparison. As can be seen in Figure 5, all composite mixtures of both curing ages exhibited strain-hardening-like behavior, showing their intrinsic tensile properties. The formation of cracks intensified with increased stress increment. After attaining the maximum strength, all the ECC specimens experienced localized failure. Overall, the tensile strength showed little improvement with the addition of slag, regardless of the curing time. However, the slag-free composite (SF-0-60) showed a better fiber–matrix interface. Consequently, the experimental data indicate that partial replacement of fly ash with slag resulted in a reduction in ductility in terms of tensile strain failure. However, the extent of slag replacement by fly ash was limited in this experiment, the overall reduction in tensile strain was not significant. 

On day 28, slag-containing composite mixtures SF-5-55 and SF-15-45 exhibited a certain reduction in tensile strength failure as compared to SF-0-60, which contained fly ash only. However, the results for specimens SF-10-50 show a small increase. This may be due to having an anomalous fiber–matrix arrangement. In general, the reduction in tensile strain in high-slag replacement specimens (SF-15-45) can be attributed to the development of enhanced cementitious material hydration causing a strong bonding at the composite–fiber interface due to slag replacement. The strong bond between the fiber and the matrix restricts the slipping of fiber in the fiber composite materials and can accelerate fiber rupture, causing a reduction in tensile strain [47]. 

As a result, it appears that slag can be used in ECC mixtures as a partial replacement for fly ash without significantly affecting the ductility with a given replacement percentage.

As shown in Figure 5, the experimental results of this study show a strain capacity of 3.5% for SF-5-55, which is similar to results reported by Xu et al. [48], who used a cement-bound aggregate in fly-ash-based ECCs. In contrast, the values for SF-10-50 and SF-0-60 were approximately 2.9 and 2.0%, respectively, similar to the values of SF-15-45. From the result, we can directly understand the effect of slag replacement on the tensile properties of slag/fly-ash-based ECC composite system. 

**Figure 6:**
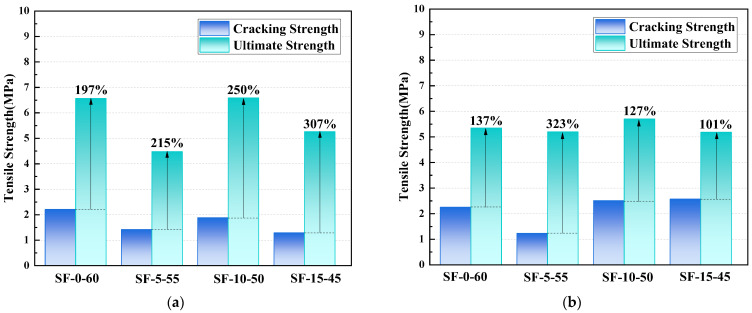
Tensile cracking and ultimate strength of SF-0-60, SF-5-55, SF-10-50, and SF-15-45. (**a**) 7 d curing, (**b**) 28 d curing.

Figure 6 summarizes the tensile cracking and ultimate strengths of the SF-0-60, SF-5-55, SF-10-50, and SF-15-45 composite specimens. The corresponding ultimate tensile strengths increases by 137%, 323%, 127%, and 101%, respectively, when compared with the first cracking strength at 28 days, indicating the existence of a noticeable amount of strain-hardening behavior. The strain-hardening behavior was reduced with increasing slag content and longer curing time, with the exception of SF-5-55. This slag/fly-ash-based composite showed a certain deviation from the remaining groups, which might be the result of having different fiber distributions and hydration processes, although this needs to be investigated further. 

**Figure 7:**
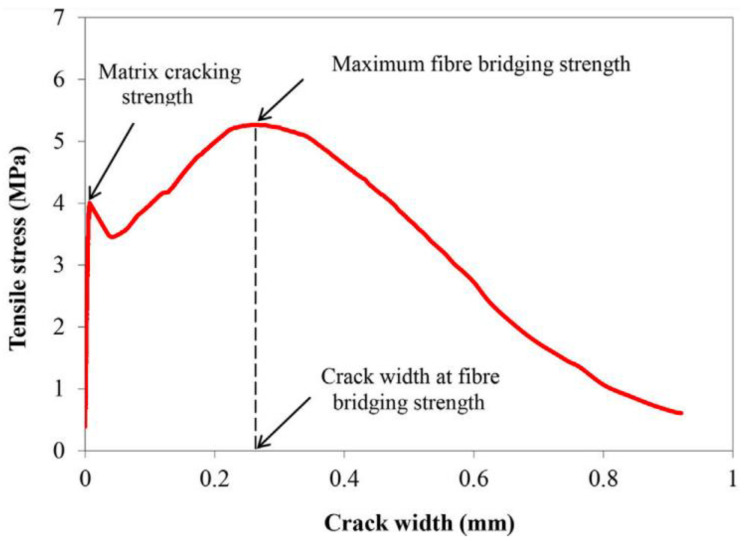
Fiber-bridging stress–crack opening curves of the composites obtained from uniaxial tensile tests with notched dog-bone-shaped specimens (Reprinted/adapted from Lan et al.) [7].

The crack opening at maximum fiber bridge strength ($\delta_{0}$) is a parameter often used to define fiber-bridging properties [48]. The applied tensile load is transferred from matrix to fiber within the fracture through the fiber–matrix interface once the ECC composite starts cracking under tension load. When the crack width is beyond the maximum fiber bridge strength ($\delta_{0}$), fibers are generally pulled out of the matrix, the load-carrying capacity of the interface is reduced, and the overall composite interface is affected, as shown in Figure 7. Therefore, all the cracks in an ECC composite matrix should be smaller in size than the maximum fiber bridge strength ($\delta_{0}$) in order to obtain a higher tensile ductility. These phenomena also represent the strain-hardening process and multiple-cracking behavior of composite materials. 

The number of cracks and the average crack width of slag/fly-ash-based ECCs after exceeding their ultimate tensile strain was measured from digital photographs. SF-15-45 had the largest crack widths, as well as the largest number of cracks compared to the other three mixes, indicating a poor crack-control ability. Details of the crack width analysis will be presented in Section 3.4. 

### 3.3. Flexural Bending Strength

**Figure 8:**
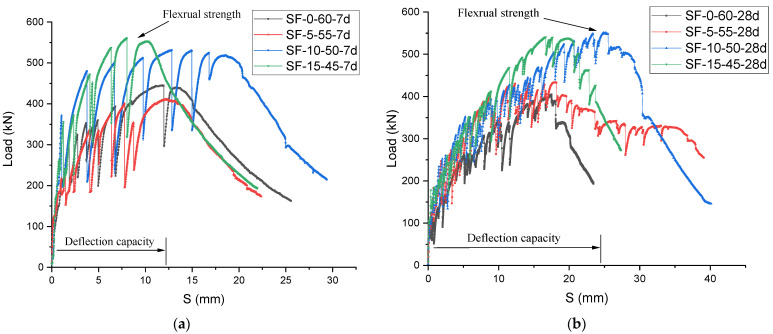
Deflection–flexural strength curves for slag/fly-ash-based ECC mixtures at: (**a**) 7 days (**b**) 28 days.

The flexural strengths of the 7- and 28-day composite specimens with varying percentages of slag and fly ash for are shown in Figure 8a,b. Generally, given the same cement and fiber content, the slag/fly-ash-based composites with 10% slag (SF-10-50) exhibited slightly higher flexural strength than the fully fly-ash-based ECC composite, whereas the specimens containing 15% slag/fly-ash-based composite (SF-15-45) exhibited a slight reduction in flexural strength compared to the SF-10-50 slag/fly-ash-based ECC. As the slag content increased from 5% to 15% by volume, the flexural strength of the slag/fly-ash-based composite with PE fiber first increased and then decreased within the range of the 403.48 to 553.48 kN for the former and from 553.4 to 543.12 kN for the latter. However, curing time had no obvious effect on the flexural strength of the slag-free ECC composite. Consequently, it appears that incorporating slag into a fly-ash-based ECC composite matrix can improve deflection of ECCs by up to 65% [11]. 

The first cracking strengths of SF-15-45 and SF-10-50 were higher than that of SF-0-60 after a 28-day curing period due to the low strength of SF-0-60. In addition, the cracking strength of SF-15-45 was slightly higher than the cracking strength of SF-10-50. This may be due to different fiber–matrix interfaces. ECC composite mixture SF-0-60 showed low tensile crack strength compared to SF-15-45 as a result of the lower fracture toughness of SF-0-60. These results are similar to those reported in other studies showing that the ultimate strength increases with increasing slag content (e.g., Zhu et al., 2012 [11]). 

### 3.4. Crack Characteristics of Loaded ECC

**Figure 9:**
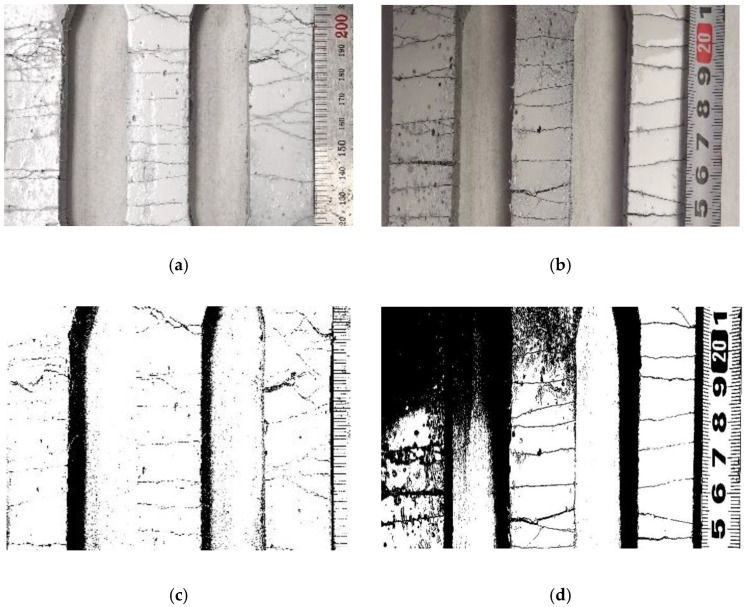

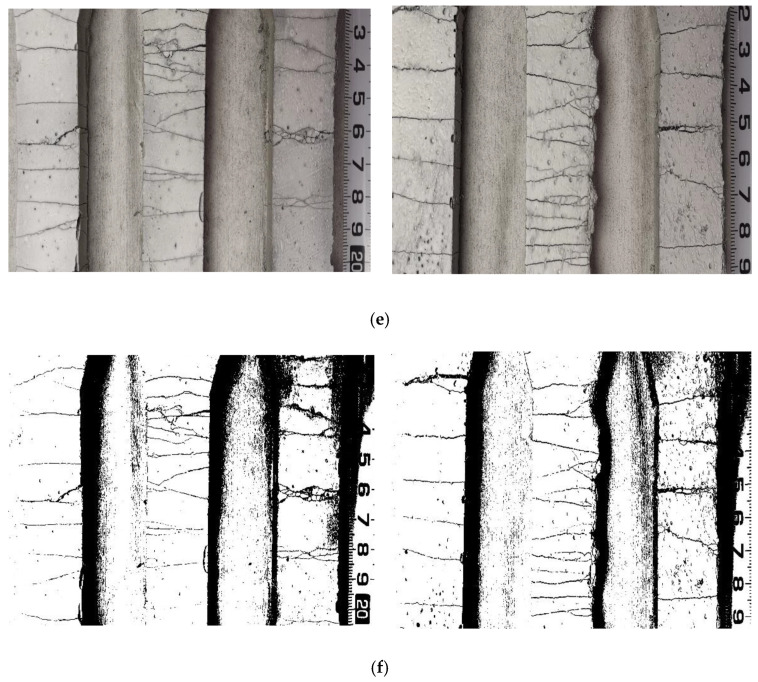
Damage pattern at 28 d in (**a**) (0%-Slag)/ SF-0-60, (**b**) (5%-Slag)/ SF-5-55, (**c**) (10%-Slag)/ SF-10-50, (**d**) (15%-Slag)/SF-15-45, and (**e**,**f**) corresponding a binary image of crack path.

Digital images of the cracking patterns in the central part of the dog-bone specimens were captured and analyzed by Image J software, a Java-based image analysis program. Figure 9 shows the crack patterns of the SF-0-60, SF-5-55, SF-10-50, and SF-15-45 ECC specimens under direct tensile loading. In order to make a comparison of cracking patterns of various composites after reaching ultimate tensile strength, three dog-bone specimens were used for analysis. All ECC specimens showed noticeable multi-cracking behavior and an increased number of cracks as the strain level increased. The SF-15-45 slag/fly-ash-based ECC matrix exhibited the most pronounced multiple-cracking behavior and the largest flexural deformation capacity among the four ECC matrices. 

**Figure 10:**
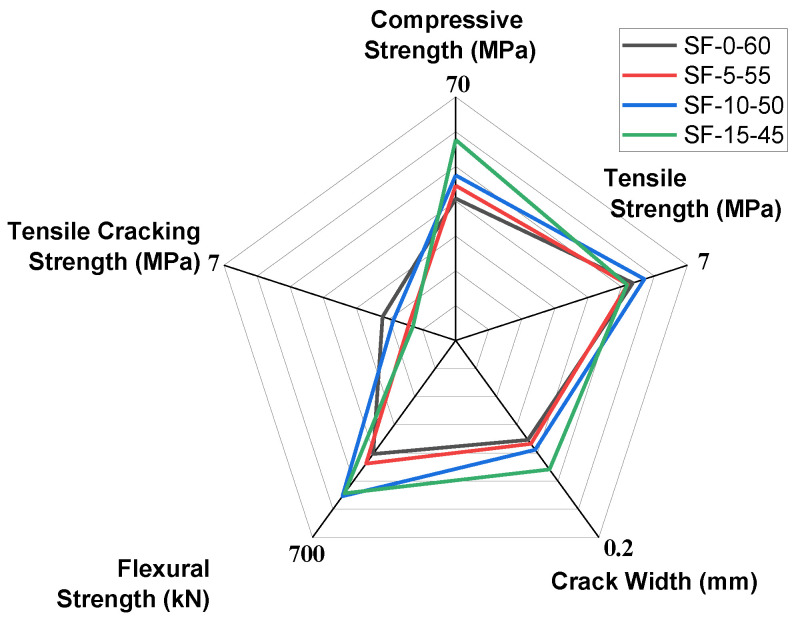
Multi-parameter assessment of mechanical and cracking characteristics of slag/fly-ash-based ECCs (SF-0-60, SF-5-55, SF-10-50, and SF-15-45).

With increasing slag content, concentrated cracking behavior appeared, as shown in Figure 9. The number of cracks increased, and the crack widths of the composite materials slightly increased with increased slag content. As inferred from Table 4, the higher fracture toughness of slag/fly-ash-based ECCs might result in a low ultimate tensile strength of the composite matrix. In contrast, as shown in Figure 10, the low fracture toughness of slag-free ECCs might result in high tensile strength, with enhanced multiple-cracking behavior and tensile ductility. 

In view of the mechanical properties and crack characteristics of slag/fly-ash-based composites, a five-parameter assessment technique was used, as shown in Figure 10. For evaluation of the mechanical properties, tensile cracking strength, compressive strength, tensile strength, flexural strength, and average crack width were taken as factors. SF-15-45 showed the highest compressive and flexural strength, and SF-0-60 showed the highest tensile strength capacity. In addition, due to the low range of slag concentrations, there was little variation in the tensile strength and tensile cracking strength. However, the compressive and flexural strength of SF-15-45 and SF-10-50 were much higher than those of SF-5-55. Therefore, the overall performance of SF-10-50 was better when all five factors are considered. In practice, a small average crack width is more appropriate for ECCs [48]. In addition, there is good association between the compressive strength value and the flexural deflection capacity from the three-point bending test. 

### 3.5. Mineralogical and Microstructural Characterization

#### 3.5.1. SEM and EDS

**Figure 11:**
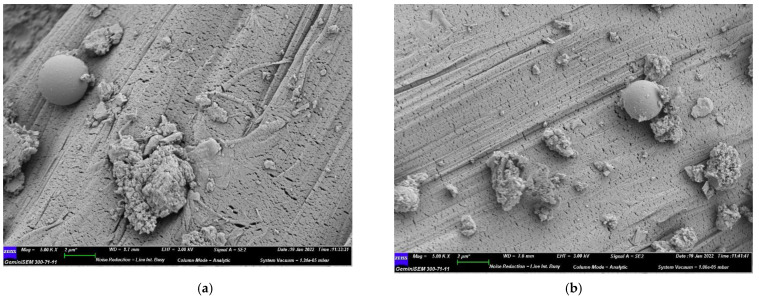

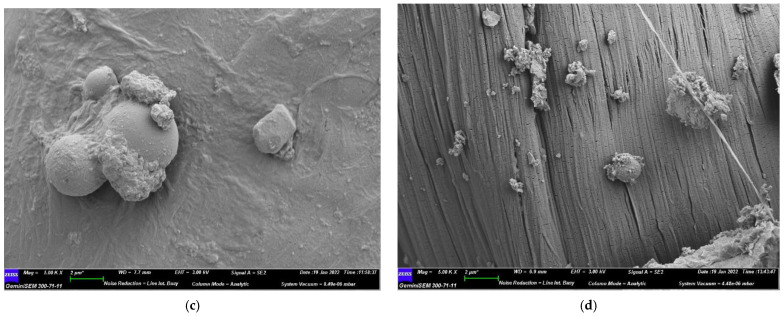
SEM results on day 7. (**a**) SF-0-60, (**b**) SF-5-55, (**c**) SF-10-50, (**d**) SF-15-45.

**Figure 12:**
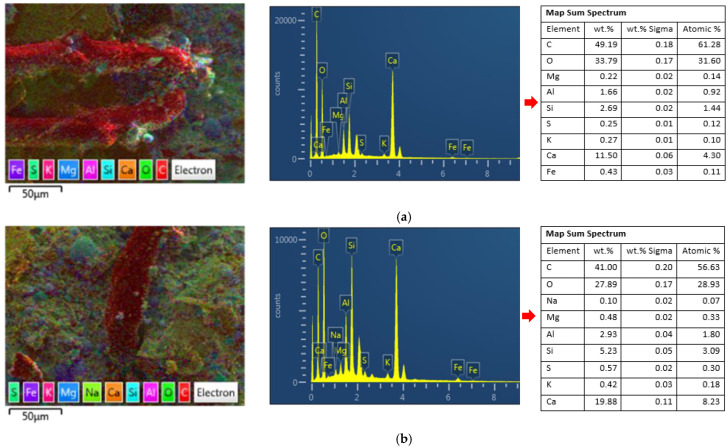

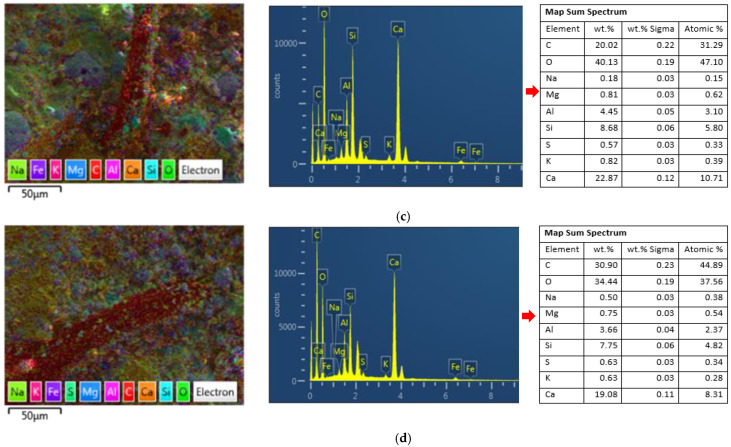
Mapping analysis of EDS spectra for 7-day specimens. (**a**) SF-0-60, (**b**) SF-5-55, (**c**) SF-10-50, (**d**) SF-15-45.

The presence of fly ash resulted in a somewhat porous structure, as shown in Figure 11. The fly ash particles reduced the interfacial crosslinking characteristics and compromised the friction bond between PE fibers and the hardened matrix during the hydration process, resulting in a good ductility of the ECC matrix. In the mixture containing fly ash and a lesser amount of slag, the fiber and the cementitious matrix showed comparatively better linkage. The introduction of slag significantly increased the extent of the cementitious matrix, leading to the presence of a high-density interface in between the aggregate and cement paste. This process can be observed in Figure 11d. Figure 11c,d shows that the chemical reaction of the slag with the calcium hydrates and other hydration byproducts tends to result in a less porous matrix microstructure. The typical spherical shape of fly ash, which has a ball-bearing effect, enhances the flow characteristics of the fresh composite matrix and improves the distribution of fiber in the composite matrix during the mixing process. Figure 11a–c also displays the positive effect of fly ash with respect to having a uniform fiber distribution compared to slag. In the mixture containing fly ash and a lesser amount of slag, the fiber and the cementitious matrix showed comparatively better linkage, as seen in Figure 11 and Figure 12, resulting in a reduction in the strain-hardening behavior and enhanced strength during the loading stage. 

Two EDS analyses were performed for each specimen: elemental analysis and mapping analysis (Figure 12). The EDS elemental analyses showed that the surfaces of the composite containing fly ash and slag exhibited the presence of oxygen (O), silica (Si), aluminum (Al), sulfur (S), calcium (Ca), magnesium (Mg), and trace amounts of iron (Fe). The data also show that the ratios of the elements vary due to the differences in slag concentrations in the composite matrix. The ratio of calcium to silica in the cementitious matrix was determined using EDS. Past studies have shown that CaCO_3_ and C-S-H/Ca (OH)_2_ were the leading phase for refining and restoring cracks [46]. 

The Ca/Si ratio in the cementitious matrix varies for each mixture with the percentage of slag replacement. The Ca/Si ratios for the slag-containing mixtures SF-5-55, SF-10-50, and SF-15-45 were 3.80, 2.60, and 2.46, respectively. However, as shown in Figure 12a–d, the ratio for the slag-free cementitious composite was 4.27. This high value indicates that higher concentrations of C-S-H occur in the slag-free cementitious composite compared to cementitious composites with high slag contents at short curing times. However, these test results contradict those reported by Tahmouresi et al. [46], who explicitly mentioned that changes in slag content have a noticeable positive effect on the Ca/Si ratio compared to the fly-ash-based cementitious composite. After 28 days of curing, XRF analysis showed that the Ca/Si ratio for all the composite mixtures was relatively similar, probably due to the prolonged hydration reaction in slag cement.

The concentrations of silicon (Si) (light blue color in the EDS images) in the slag-based composite matrix were relatively higher than in the slag-free fly-ash-based cementitious composite. This indicates the presence of an interfacial transition zone (ITZ) of aggregate and cement paste. In addition, the concentration of calcium (Ca) (orange color) in the purely fly-ash-based cementitious composite was relatively higher than that in the slag-based cementitious composites, indicating that calcium silicate hydrate gel (C-S-H) is the main constituent. In addition, the occurrence of Ca (orange color) without the appearance of other elements, such as Si, Fe, and Al, in all composite mixtures represents the crystalline phase of calcium hydroxide (C-H) in the cementitious composite. The Ca/Si ratio in the results of XRF analysis showed that ratios were notably lower in the hydrated silicate and aluminate, possibly because of the prolonged hydration activity of the slag-based cementitious composite. 

**Figure 13:**
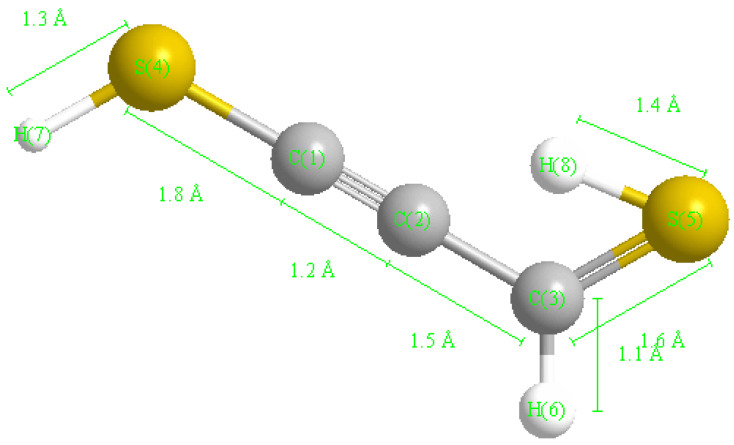
Single-crystal structure.

The hydration of pure fly ash was slow, and only a less amorphous and gelatinous component adheres to the ECC matrix, according to the EDS mapping analysis of spectrum and XRD results of sample SF-0-60. Because this amount of gel could not really fill the holes between the hydration products, the microstructure of SF-0-60 was loose, and compressive strength did not increase significantly. The addition of 15% slag to the ECC matrix notably increased compressive strength at 28 days (40.79–55.05 MPa). CaO (C) in slag reacted with water (H) to form Ca (OH)_2_ (CA) and then C-S-H and C-A-S-H, which enclosed the particles and filled the pores, as indicated by the single-crystal structure and Equations (1) and (2), respectively (Figure 13) This resulted in good compressive strength at 28 days, as shown by the results for sample SF-15-45.

#### 3.5.2. XRD and XRF Analysis

**Figure 14:**
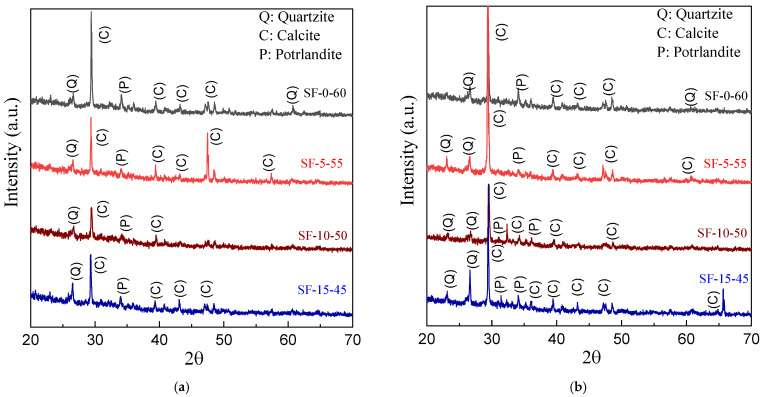
XRD results for 7 and 28 days. (**a**) 7 d, (**b**) 28 d.

The chemical reaction of Portland cement with water results in the formation of various hydration products. In order to proceed, a pozzolanic reaction resulting in the formation of portlandite is mandatory [27]. The presence of pozzolanic materials may result in portlandite forming additional hydration products similar to those formed during cement hydration. Both XRD and XRF were used to quantitatively study the formation of hydration products, and the results are shown in Figure 14 and Table 5. The reduction in unbound calcium hydroxide improves the chemical stability of the ECC [49,50].

The major components of Portland cement are CaO, SiO_2_, and Al_2_O_3_; other constituents include C_3_S, C_2_S, C_3_A, and C_4_AF, where C: CaO, S: SiO_2_, A: Al_2_O_3_, and F: Fe_2_O_3_ (Table 5). The main constituents occurring in the microstructures of hydrated cement paste include calcium silicate hydrate (C–S–H), calcium hydroxide (C–H), ettringite (Aft), monosulfate (AFm), unhydrated (UH) cement particles, and air voids [51], with calcium silicate hydrate (CSH) being the principal solid component. This is formed by the hydration of calcium silicates. The reactions of C_3_S and C_2_S may be expressed as:

(8) $$2C_{3}S+6H\to C_{3}S_{2}H_{3}+3CH$$

(9) $$2C_{2}S+4H\to C_{3}S_{2}H_{3}+CH$$

The reaction of the aluminate phase is rapid and can cause a short setting time. Consequently, roughly 5% gypsum (calcium sulfate) was added to Portland cement to control the cement hydration reaction rate. The hydration reaction of C_3_A with gypsum and water is as follows [28]:

(10) $$C_{3}A+3CSH_{2}+26H\to C_{3}A.3CS.32H$$

In this reaction, S represents SO_3_. Ettringite, the end product of the reaction, forms prismatic crystals on the surfaces of C_3_A and subsequently reacts with C_3_A to form calcium monosulfate. C_4_AF hydrates in a similar manner to C_3_A but at a slower rate. Research on these pure phases assists in understanding the hydration process, as it is complicated due to the interaction of phases and minor constituents [28]. The solid-state molecular structure of the hydration process is presented in Figure 13. 

Figure 14 presents XRD spectra of slag/fly-ash-based ECCs with varying amounts of slag replacement at 7- and 28-day curing ages. The main crystalline mineral phases in the slag/fly-ash-based cementitious composites were quartzite (SiO_2_), portlandite (Ca (OH)_2_), and calcite (CaCO_3_). However, a small quantity of gypsum (CaSO_4_.2H_2_O) and ettringite (Ca_6_Al_2_ (SO_4_)_3_(OH)_12_.26H_2_O) were also detected, as shown in Figure 14a,b. The diffraction peaks of calcite and portlandite increased with increasing curing period, as shown in Figure 14, and the diffraction peak for calcite was the highest for the slag-free fly-ash-based ECC at the shorter age of curing. In addition, the ECC matrix with 5% slag replacement (SF-5-55) showed a slight deviation in diffraction peaks at the longer curing time based on the XRD and XRF results, supporting the previous discussion in Section 3.3. Flexural Bending Strength.

## 4. Conclusions

In this paper, we presented the effect of various slag replacements on the compressive and tensile properties, as well as the microstructural and mineralogical characteristics of slag/fly-ash-based ECCs. Based on the experimental results and materials used, the following conclusions can be made:

The slag-based cementitious matrix had a more highly cross-linked and dense microstructure at the matrix–aggregate interface. In addition, the concentration of particles on the surface of the fibers was higher in the slag-containing cementitious composites than in the fly-ash-based mixtures. This fact supports the concept that there is a stronger bonding between the matrix and fibers in slag-based cementitious matrices than fly-ash-based matrices. According to our experimental study and microstructural analysis, the higher concentration of slag in SF-15-45-ECC resulted in greater crack widths under the same tensile strain compared to the other specimens. The overall performances of SF-5-55 and SF-10-50 were the best of the slag/fly-ash-based composite mixes studied. When the amount of raw material (slag) is considered, SF-10-50 is recommended for practical applications.

Slag can be used in the production of ECC mixtures as a partial replacement for fly ash without significantly affecting the ductility at a given replacement percentage. However, the strong bond between the fiber and the matrix restricts the slipping of fiber in fiber composite materials and can accelerate fiber rupture, causing a reduction in tensile strain. In summary, the microstructural, mineralogical, and mechanical properties of slag/fly-ash-based ECCs were properly studied under a normal environmental exposure in the present study. However, the study of these properties can be further improved in the future by considering various environmental exposure conditions, curing temperatures, the presence of and cracks. In addition, the impact of fiber distribution and the slag/fly ash replacement range should be considered. Ignoring the impact of fiber alignment and distribution in composite materials could lead to a large error that contradicts the structural application of ECCs.

## Figures and Tables

**Table 1:** Mix proportions of ECCs (kg/m^3^).

Mix ID	Slag	FA	Cement	Water	Sand	PE	HRWR
**SF-0-60**	0.00	799.90	533.30	400	266.60	15	4
**SF-5-55**	66.60	733.20	533.30	400	266.60	15	4
**SF-10-50**	133.30	666.60	533.30	400	266.60	15	4
**SF-15-45**	199.90	599.90	533.30	400	266.60	15	4

**Table 2:** Chemical composition (X-ray diffraction) of fly ash, slag, and cement.

	SiO_2_	Al_2_O_3_	Fe_2_O_3_	SO_3_	CaO	MgO	Na_2_O	K_2_O	Others	LOI ^1^
**Fly ash (FA)**	45.20	26.10	6.93	1.38	11.84	1.28	1.73	1.54	6.64	4.32
**Slag (SL)**	36.50	14.30	3.20	2.21	33.60	7.50	0.38	0.34	0.80	0.46
**Cement (C)**	17.94	4.46	3.56	3.19	64.56	3.75	0.17	1.17	0.30	3.01

^1^ Loss of ignition (LOI).

**Table 3:** Properties of PE fiber.

Name	Fiber Length (mm)	Diameter (µm)	Young’s Modulus (GPa)	Elongation (%)	Tensile Strength (MPa)	Density (g/cm^3^)
**PE**	18	20	100	3	3000	0.97

**Table 4:** Mechanical properties of slag/fly-ash-based ECCs.

Specimen Designation	First-Crack Tensile Strength (MPa)		Ultimate Tensile Strength (MPa)		Ultimate Flexural Load (kN)	
	7 Days	28 Days	7 Days	28 Days	7 Days	28 Days
**SF-0-60**	2.21	2.25	6.57	5.35	445.70	403.48
**SF-5-55**	1.42	1.23	4.48	5.20	411.30	438.00
**SF-10-50**	1.88	2.51	6.59	5.70	531.90	553.48
**SF-15-45**	1.29	2.57	5.26	5.18	561.50	543.12

**Table 5:** XRF results of slag/fly-ash-based ECC at 28 days.

Compound (c)	Element (E)	(c) wt.%	(E) wt.%	(c) (wt.%)	(E) (wt.%)	(c) (wt.%)	(E) (wt.%)	(c) (wt.%)	(E) (wt.%)
		**SF-0-60**		**SF-5-55**		**SF-10-50**		**SF-15-45**	
**CaO**	Ca	40.04	28.63	36.80	26.31	39.68	28.37	40.23	28.77
**SiO_2_**	Si	32.90	15.38	34.50	16.13	32.77	15.32	33.20	15.52
**Al_2_O_3_**	Al	16.79	8.89	18.08	9.57	16.90	8.94	16.10	8.52
**Fe_2_O_3_**	Fe	4.23	2.96	4.21	2.94	3.89	2.72	3.76	2.63
**MgO**	Mg	1.75	1.06	2.01	1.21	2.33	1.41	2.51	1.51
**SO_3_**	Sx	1.72	0.688	1.67	0.668	1.66	0.666	1.61	0.645
**K_2_O**	K	1.06	0.876	1.20	0.993	1.14	0.944	1.09	0.903

## Data Availability

Not applicable.
